# Pseudo Multi-Modal Approach to LiDAR Semantic Segmentation

**DOI:** 10.3390/s24237840

**Published:** 2024-12-08

**Authors:** Kyungmin Kim

**Affiliations:** School of Integrated Technology, Yonsei University, Incheon 21983, Republic of Korea; kyungmin.kim@yonsei.ac.kr

**Keywords:** LiDAR semantic segmentation, knowledge distillation

## Abstract

To improve the accuracy and reliability of LiDAR semantic segmentation, previous studies have introduced multi-modal approaches that utilize additional modalities, such as 2D RGB images, to provide complementary information. However, these methods increase the cost of data collection, sensor hardware requirements, power consumption, and computational complexity. We observed that multi-modal approaches improve the semantic alignment of 3D representations. Motivated by this observation, we propose a pseudo multi-modal approach. To this end, we introduce a novel class-label-driven artificial 2D image construction method. By leveraging the close semantic alignment between image and text features of vision–language models, artificial 2D images are synthesized by arranging LiDAR class label text features. During training, the semantic information encoded in the artificial 2D images enriches the 3D features through knowledge distillation. The proposed method significantly reduces the burden of training data collection and facilitates more effective learning of semantic relationships in the 3D backbone network. Extensive experiments on two benchmark datasets demonstrate that the proposed method improves performance by 2.2–3.5 mIoU over the baseline using only LiDAR data, achieving performance comparable to that of real multi-modal approaches.

## 1. Introduction

LiDAR semantic segmentation aims to assign category labels at the point level, enabling a spatial and contextual understanding of complex visual 3D scenes. It is widely adopted in various applications such as autonomous driving, robotics, and remote sensing, where high standards of spatial precision and contextual awareness are critical [1,2,3].

To improve the performance, multi-modal approaches leveraging complementary information from multiple sensors have been proposed. Specifically, combining the rich visual cues, such as color and texture, from RGB images with the precise depth perception and geometric structure provided by LiDAR has become a popular strategy for various LiDAR-related tasks [4,5,6,7,8]. While these methods enhance accuracy and reliability, they require paired data of LiDAR point clouds and camera images during both training and inference, enforcing strict point-to-pixel alignment–a significant limitation for practical deployment. The acquisition and processing of multi-modal data increase the cost of data collection and computational demands. In practice, these approaches also raise vehicle sensor installation expenses and in-vehicle power consumption during operation. Moreover, when sensors across modalities differ in resolution and viewpoint, those points with complete data across all modalities can be utilized in fusion-based approaches, limiting the usable data to a subset of the total available input.

**Figure 1:**
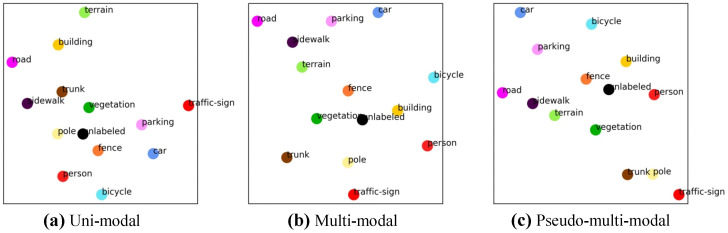
Visualization of 3D feature distribution across different approaches. (**a**) Uni-modal, (**b**) real multi-modal, and (**c**) proposed pseudo multi-modal LiDAR semantic segmentation frameworks. The real multi-modal method and proposed pseudo multi-modal method exhibit better semantic alignment compared to the uni-modal method, as evidenced by closer distances between class features within the same super-category. The colors for each class in the figure follow the official colormap of SemanticKITTI.

**Figure 2:**
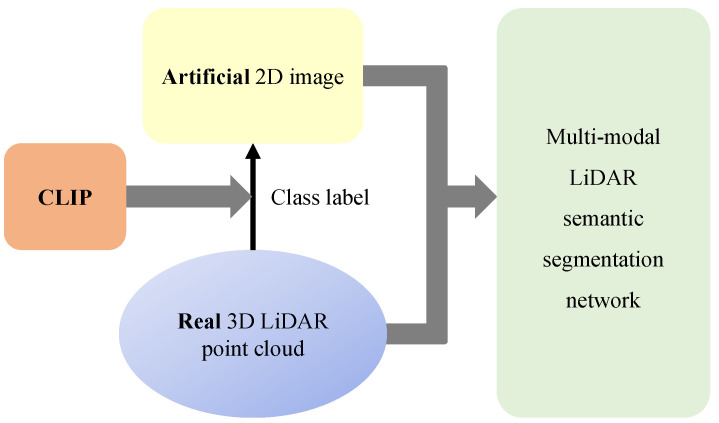
Overview of proposed pseudo multi-modal LiDAR semantic segmentation framework. Using a vision–language model (e.g., CLIP), we generate pseudo RGB images aligned with LiDAR data. These LiDAR and pseudo RGB pairs are used to train a multi-modal segmentation model.

To enhance LiDAR semantic segmentation performance by efficiently utilizing uni-modal data, we propose a novel pseudo multi-modal approach. As shown in Figure 1, we observe that multi-modal approaches improve the semantic alignment of 3D representations. Motivated by this observation, we synthesize artificial 2D feature to convey semantic information to 3D features. Leveraging the close semantic alignment between image and text features within the shared embedding space of vision–language models, we synthesize artificial 2D feature by arranging text features of class labels. These artificial 2D features transfer semantic knowledge to 3D features through knowledge distillation and enabling consistent relationships across various classes. Figure 2 visualizes the brief concept of proposed pseudo-multi-modal framework. Furthermore, we conducted extensive prompt design experiments to improve class distinction and effectively capture semantic relationships.

Using the artificial 2D features generated by our method, we trained a pseudo multi-modal LiDAR semantic segmentation model on the SemanticKITTI and nuScenes benchmarks. Our model achieved 97–99% of the performance of conventional multi-modal methods that utilize both RGB and LiDAR, while requiring only uni-modal LiDAR data. This demonstrates the successful generation of artificial 2D images and the effective distillation of semantic information into the LiDAR segmentation network without the need for additional modality data. The proposed pseudo multi-modal framework, which eliminates the need for additional modality data during both training and inference, substantially reduces data acquisition costs and computational overhead. This unique design makes it highly practical for diverse scenarios.

In summary, the contributions of this paper are as follows:

- We introduce a pseudo multi-modal LiDAR segmentation framework based on a novel artificial 2D construction method.
- The proposed pseudo multi-modal framework achieves performance on par with real multi-modal LiDAR semantic segmentation using only uni-modal training data and uni-modal inference input on the two benchmark datasets.

## 2. Related Works

LiDAR semantic segmentation methods are typically categorized based on how they represent and process LiDAR point clouds: projection-based, voxel-based, and point-based approaches. Projection-based methods [9,10] project 3D point clouds onto a 2D plane, often converting LiDAR data into bird’s-eye view (BEV) or range images. This projection simplifies the processing by reducing dimensionality, allowing for 2D convolutional neural networks (CNNs) to be applied. Projection-based methods are computationally efficient and benefit from mature 2D segmentation techniques but can suffer from information loss due to projection, especially for detailed or small objects. Voxel-based methods [11,12] partition the 3D space into a grid of small volumetric cells, or voxels, where each voxel can contain multiple points. These methods make it easier to apply 3D convolutional operations and capture spatial relationships within a 3D space. However, voxelization introduces quantization errors, and the computational complexity can become high when finer resolutions are required for accuracy. Point-based methods [13,14] process the raw 3D points directly, without projection or voxelization. These approaches use pointwise neural networks that preserve the original spatial structure and avoid information loss. While point-based methods retain finer details, they can be computationally intensive and require specialized network architectures to handle unstructured point cloud data. Each approach is selectively employed to achieve a balance between accuracy and computational efficiency, with some methods combining multiple views to enhance performance.

On the other hand, other approaches take advantage of entirely different sensor data to improve the performance [4,5,6,7,8,15,16,17]. Among the various modalities, RGB has garnered significant attention for providing rich visual cues, such as color and texture, which complement LiDAR data. Consequently, multi-modal methods combining RGB and LiDAR have been widely adopted across numerous LiDAR-related tasks, leading to significant performance improvements. Refs. [5,6,7,8,17] utilized the complementary information between LiDAR and RGB to enhance LiDAR semantic segmentation performance. PMF [5] proposed a perception-aware multi-sensor fusion approach to mitigate the performance degradation caused by discrepancies between the two modalities by leveraging perceptual information from both. BEVFusion [7] introduced a framework that unifies multi-modal features in a bird’s-eye view (BEV) representation space to prevent the semantic density of RGB features from deteriorating due to point-level fusion between modalities. However, these fusion-based methods require both modalities as inputs during both training and inference, resulting in higher computational costs and hardware requirements. In contrast, 2DPASS [6] introduced a distillation-based framework to improve efficiency by allowing for inference with only the LiDAR modality. Nevertheless, unlike our method, 2DPASS still requires both modalities during training, which limits the usable data to points with complete information across all modalities, thereby restricting the amount of data that can be utilized.

## 3. Methods

We propose a novel pseudo multi-modal LiDAR semantic segmentation framework. Using a vision–language model (e.g., CLIP [18]), we generate pseudo RGB images aligned with LiDAR data, creating LiDAR and pseudo RGB pairs to train a multi-modal segmentation model. Our approach builds on the baseline established by 2DPASS [6], which is one of the state-of-the-art multi-modal semantic segmentation models leveraging both LiDAR point clouds and RGB images. The key contribution of our method is distilling semantic information from pseudo RGB images instead of real ones. Despite its simplicity, our approach effectively generates pseudo RGB images, achieving performance comparable to 2DPASS with real RGB inputs. In the following subsections, we describe the 2DPASS baseline, the novel artificial 2D image construction method, the text prompt design approach, and the overall training pipeline.

### 3.1. 2DPASS Baseline

The architecture of 2DPASS consists of a 2D branch and a 3D branch. Each branch consists of an encoder–decoder architecture designed to perform semantic segmentation for its respective modality input. During training, each branch receives inputs of RGB images and LiDAR point clouds, respectively. Multi-scale features are extracted from each branch’s encoder. Then, a point-to-pixel mapping is performed to enable multi-scale knowledge distillation between corresponding 2D and 3D features. This distillation process trains the 3D features to align closely with the fused features, which combine both 2D and 3D feature representations. Since the 3D features receive only distilled knowledge without fusion with other modalities, the 3D branch operates independently during inference, enabling LiDAR point cloud inference without the need for the 2D branch or RGB images. In this way, 2DPASS minimizes additional computational overhead during inference compared to fusion-based multi-modal approaches. 2DPASS effectively harnesses the advantages of multi-modal data, achieving remarkable performance.

### 3.2. Artificial 2D Image Construction

**Figure 3:**
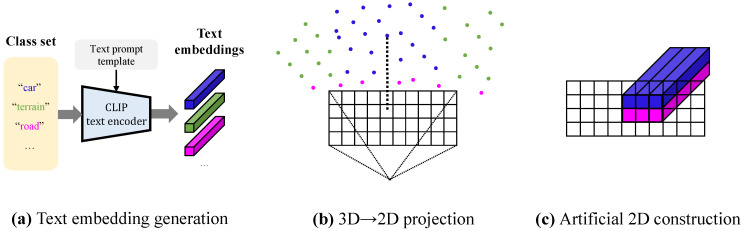
Artificial 2D image construction process. (**a**) Using the CLIP text encoder, we obtain text embeddings corresponding to each class in the dataset. (**b**) We project the 3D point cloud onto a 2D image plane, mapping each point to a corresponding (i,j) pixel. (**c**) For each (i,j) pixel where a 3D point is projected, we assign the text embedding associated with the point’s class label. By filling the entire 2D image plane in this manner, we construct an artificial 2D image. This process requires only the pre-obtained text embeddings for the class set and the 3D LiDAR point cloud. The colors for each class in the figure follow the official colormap of SemanticKITTI.

In this section, we introduce our novel pseudo-RGB image generation process. We visualize a construction process in Figure 3. First, we project LiDAR points into a 2D image plane using arbitrary camera parameters. Unlike conventional methods, which are limited to a fixed viewpoint from a finite number of real RGB cameras, our approach allows for synthetic images to be generated from multiple diverse viewpoints. In practice, we simply utilize the parameters of the real RGB camera in the actual implementation. Next, each pixel in this 2D plane is assigned a CLIP text feature corresponding to the class label of the original 3D point, forming the artificial image features. For example, if a point corresponding to the car class projects to the (i,j) position, we pass the “A photo of a {car}.” to the vision–language model’s text encoder, placing the resulting text embedding at that location. Similarly, if a point for the road class projects to the (i+1,j) position, we assign the text embedding feature for “A photo of a {road}.” at that pixel. In this manner, we fill the 2D image plane to construct the artificial 2D image. The resulting artificial image feature has dimensions h×w×d, where *h* is the height, *w* is the width of the dataset’s real RGB images, and *d* is the embedding dimension size of the vision–language model’s text encoder.

This approach leverages the shared embedding space of CLIP, where the image features and their associated class text features are closely aligned. This shared semantic embedding allows the artificial image to effectively represent the relational semantics between different classes.

### 3.3. Text Prompt Design

To ensure that the text features used in constructing the artificial 2D image features effectively convey semantic meaning, we experimented with various designs for text prompts. Each method used to obtain the final text embeddings is indicated with an asterisk *.

#### 3.3.1. Template Selection for Text Prompt

* Default template. The prompt “A photo of a {label}.” follows a sentence structure that clearly describes the content represented by the image, serving as a foundational template across various applications and generally contributing to improved model performance. Following the convention, we adopted this as our default prompt template.
**CLIP templates.** CLIP [18] ensembles 80 different context prompts, such as “A photo of a big {label}.” and “A photo of a small {label}.”, to improve zero-shot classification performance on ImageNet compared to a single baseline prompt. Following convention, we synthesized artificial 2D image features by averaging the text embeddings across 80 prompts.
**MaskCLIP templates.** To perform zero-shot semantic segmentation, MaskCLIP [19] employs a method similar to CLIP. It feeds prompt-engineered texts into the text encoder of CLIP with 85 prompt templates, such as “There is a {label} in the scene.”, and averages the resulting 85 text embeddings of the same class.

#### 3.3.2. Providing Class Hierarchy Information

In addition to leveraging multiple templates, prior observations suggest that customizing prompt text for the task (e.g., specify image type or category) can further enhance performance [18]. Following this insight, we incorporated super-class and similar class information into the templates by utilizing the dataset’s class set hierarchy.

**Specification of super-class.** We utilized hierarchical super-class information to provide additional contextual guidance. We utilized prompt templates in the following form: “A photo of a {class}, a type of {super-class}”.
* Differentiation from similar classes. We measured the cosine similarity between text prompts for different classes and observed that these prompts did not vary significantly from one another. To enhance class distinctiveness, we included information about other classes within the same super-class, explicitly clarifying that they are not similar classes. The utilized prompt template is in the following form: “A photo of a {class}, not a {similar class}”.

#### 3.3.3. Modification of Class Descriptions

Upon measuring cosine similarity between text prompts, we observed high similarity among most prompts, with the trunk class notably having lower similarity to other classes. We hypothesized that this may be due to potential ambiguity, as the trunk could be misinterpreted as the trunk of a car rather than a tree trunk. To enhance class discriminability and convey richer semantic information, we adjusted the class label representation to move beyond single-word expressions.

* Class name. We utilized the default class names defined by the dataset without any modifications.
**Synonym set.** To distinguish homographs and incorporate richer semantic information, we used synonym sets rather than single words. For each class, synonym sets were manually curated from sources including (1) ChatGPT [20], (2) WordNet [21], and (3) Wikipedia [22]. We used the average of text embeddings obtained by passing each synonym set into the CLIP text encoder as a text embedding for each class.
**Definition.** To provide more detailed descriptions, we replaced single-word labels with definition sentences for each class to obtain text embeddings. These definition sentences were manually composed using outputs from three sources: (1) ChatGPT [20], (2) WordNet [21], and (3) Wikipedia [22]. This approach allowed us to incorporate a richer semantic representation for each class.

### 3.4. Overall Training Pipeline

**Figure 4:**
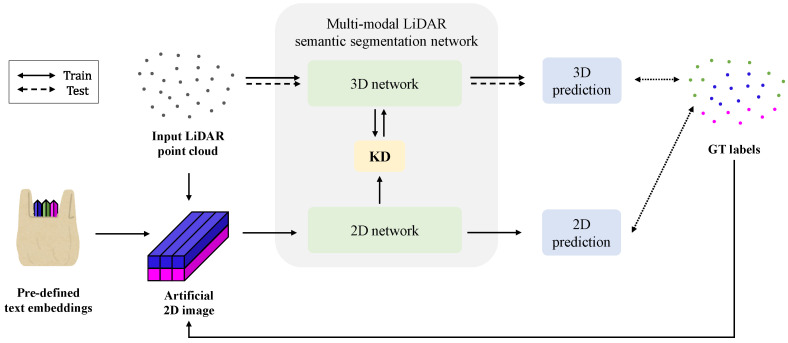
Overall training pipeline of proposed pseudo multi-modal LiDAR semantic segmentation framework. During training, we constructed an artificial 2D image from the input LiDAR data and label, forming (real LiDAR, artificial image) pairs to train the multi-modal segmentation network. During inference, only the input LiDAR passes through the 3D branch to obtain predictions for performance evaluation.

As shown in Figure 4, the overall training pipeline is as follows. First, we obtain text embeddings corresponding to the LiDAR dataset’s class set, using the most effective text prompt design (default template * differentiation from similar classes * class name). During training, when a 3D LiDAR point cloud is provided as input, we construct a corresponding artificial image as described in Section 3.2. We then train the multi-modal LiDAR semantic segmentation network using both real LiDAR input and the artificial image. Notably, our training process requires no additional data beyond the 3D LiDAR data itself and entails no additional human effort. During inference, the 3D LiDAR branch operates independently with only 3D LiDAR input, without modality fusion. Consequently, predictions are generated using the LiDAR point cloud alone, and mIoU performance is evaluated.

## 4. Experiments

### 4.1. Experimental Setup

#### 4.1.1. Dataset

Following the practice of popular LiDAR segmentation models we conducted experiments on the SemanticKITTI [23] and nuScenes [24] benchmarks. For SemanticKITTI, LiDAR data were captured by the Velodyne HDL-64E sensor, paired with corresponding frontal-view RGB images at a resolution of 1242×375. According to the official setting, sequence 08 was used for validation, sequences 00-10 (excluding 08) for training, and sequences 11–21 for testing. Pixel-wise class annotations are provided for 19 classes across the training and validation sets. For nuScenes, LiDAR data were captured by the Velodyne HDL-32E sensor, paired with 6 RGB images at a resolution of 1600×900. We followed the official split, and pixel-wise class annotations were provided for 16 classes.

#### 4.1.2. Evaluation Metric

We used mean intersection over union (mIoU) for segmentation performance evaluation. The intersection over union (IoU) is first computed for each class as the ratio of correctly predicted pixels (intersection) to the total pixels belonging to either the predicted or ground truth class (union). $\mathrm{IoU}=\frac{\mathrm{True}\;\mathrm{Positives}}{\mathrm{True}\;\mathrm{Positives}+\mathrm{False}\;\mathrm{Positives}+\mathrm{False}\;\mathrm{Negatives}}$. The mIoU is then obtained by averaging these IoU values across all classes. Higher mIoU values indicate more precise segmentation results.

#### 4.1.3. Implementation Details

We followed the setup of a previous work [6]. For the 2D backbone, we used a ResNet34 [25]-FCN [26] architecture, while for the 3D backbone, we utilized an SPVCNN [27] structure with SparseConvNet [28]. For the 3D input, we applied common data augmentation strategies for semantic segmentation, including global scaling with a random factor sampled from the range [0.95, 1.05] and global rotation around the Z-axis by a random angle. For the SemanticKITTI validation set, we trained our model with a batch size of 8 and a learning rate of 0.24 over 64 epochs using the SGD optimizer, the same as previous work for a fair comparison.

### 4.2. Experimental Results

#### 4.2.1. Ablation Study

Here, we first analyze the effect of various text prompt designs discussed in Section 3.3. Subsequently, we describe the final results obtained using the optimal combination identified from these experiments. All ablation studies were conducted using the SemanticKITTI dataset.

**Figure 5:**
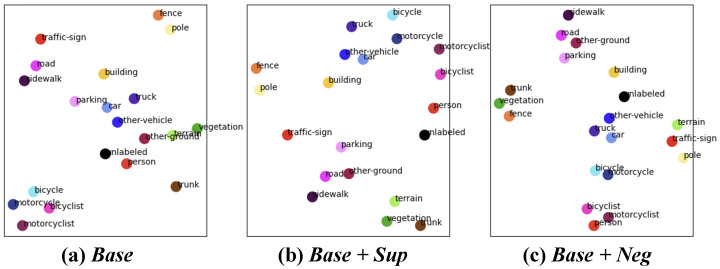
The distribution of the text embeddings for the SemanticKITTI class set. These results visualize the text embeddings obtained using each of the following text prompt templates. (**a**) *Base*: Provides only the default class name, (**b**) *Base + Sup*: Includes super-class information, (**c**) *Base + Neg*: Explicitly clarify that it is not a similar class. These tSNE plots demonstrate that providing additional information brings semantically similar classes closer together.

**Effect of Text Prompt Template Selection. **Table 1 reports the performance on the SemanticKITTI validation set for the template selection methods discussed in Section 3.3.1. We compare a single default template, CLIP templates [18], and MaskCLIP templates [19]. While prompt ensembling enhanced performance in zero-shot classification, it was not effective for distilling semantic information within our pseudo multi-modal LiDAR semantic segmentation framework. We hypothesize that templates that are not suitable for representing driving scene images are included in the conventional template set, adversely affecting the averaged text embedding.
**Effect of Class Hierarchy Information.** We conducted experiments to investigate the effect of class hierarchy information in the text embeddings that are used to construct artificial images in the proposed framework. As discussed in Section 3.3.2, we compared three prompt templates: (1) *Base*: Provides only the default class name (“A photo of a {label}.”); (2) *Base + Sup*: Includes additional information about the super-class (“A photo of a {class}, a type of {super-class}.”); and (3) *Base + Neg*: Explicitly clarify that it is not a similar class (“A photo of a {class}, not a {similar class}.”). Figure 5 visualizes the t-SNE plots of text embeddings for the SemanticKITTI class set generated using these three methods. The t-SNE plots demonstrate that providing additional information brings semantically similar classes closer together. For instance, with additional information, the distance between bicyclist, motorcyclist, and person decreases. Table 2 demonstrates that the inclusion of hierarchy information increases mIoU. We hypothesize that the high performance of Base + Neg is due to the additional class information, which strengthens associations within the same higher-level class, effectively functioning as if super-category information were also provided.
**Effect of Class Descriptions.** We adjusted the method of representing class labels to convey richer semantic information in Section 3.3.3. Table 3 reports the SemanticKITTI performance with the following three class representation methods: (1) the default class name, (2) a synonym set, and (3) class definition sentences. We observed performance improvements in classes such as traffic sign, person, and bicyclist when using synonyms or definition sentences. However, the overall mIoU showed no significant change. Given the minimal performance gain relative to the additional human effort required, we opted to retain the default class names. Nevertheless, certain datasets or specific classes may benefit from alternative descriptions for improved accuracy.
**Effect of camera parameter.** We modified the artificial 2D construction process to use random camera parameters among all available camera viewpoints in the dataset, instead of using ground-truth camera parameters. It achieved a 67.1 mIoU and also outperformed the baseline performance shown in Table 4. This result confirms that the artificial features effectively convey semantic information regardless of specific viewpoints.

#### 4.2.2. Main Results

**Figure 6:**
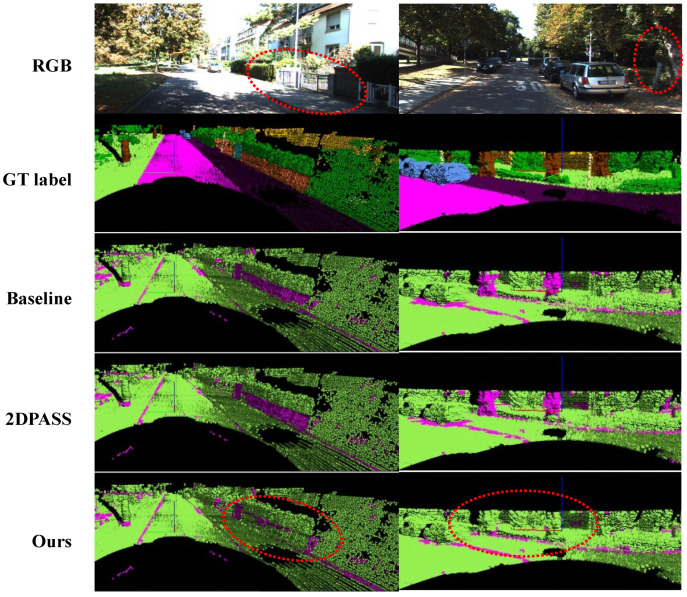
Qualitative examples of segmentation results on the SemanticKITTI validation set. From top to bottom, the figure visualizes the RGB image, ground truth, and results for baseline, 2DPASS, and ours. Each point in the ground truth is colored using the official SemanticKITTI colormap. In the bottom three rows, green points indicate correct predictions, while magenta points represent incorrect predictions. The red dashed circles highlight the differences between predictions. The proposed method achieves more accurate segmentation for thin objects such as tree trunks and fences.

**Figure 7:**
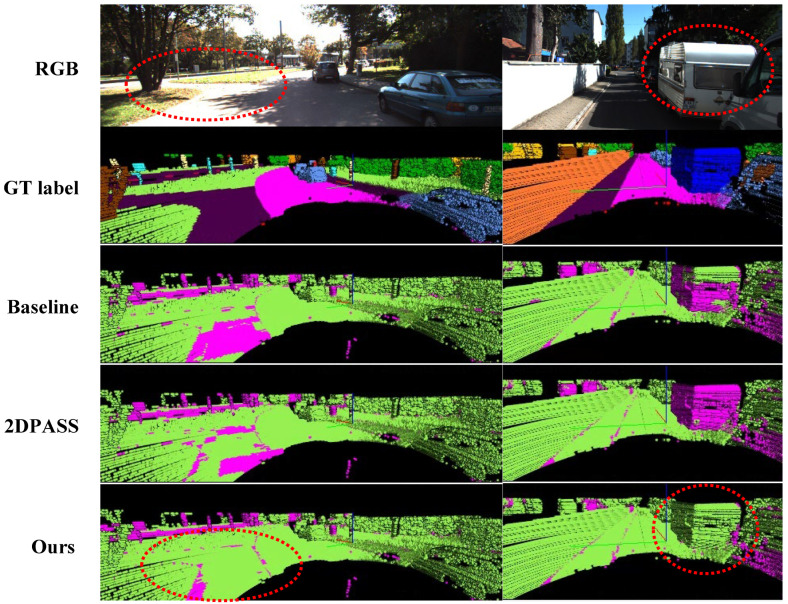
Additional qualitative examples of segmentation results on the SemanticKITTI validation set. From top to bottom, the figure visualizes the RGB image, ground truth, results for baseline, 2DPASS, and ours. Each point in the ground truth is colored using the official SemanticKITTI colormap. In the bottom three rows, green points indicate correct predictions, while magenta points represent incorrect predictions. The red dashed circles highlight the differences between predictions. The proposed method achieves more accurate predictions in challenging cases where confusion may occur within the same super-category.

For quantitative evaluation, we compared the mIoU performance of the baseline, 2DPASS, and our proposed method. The baseline results refer to the uni-modal experiment using the same 3D backbone. Both ours and 2DPASS employ the same architecture and a four-scale knowledge distillation setting. The baseline and 2DPASS results were reproduced using the official code. Neither additional fine-tuning nor test-time augmentation was applied. As shown in Table 4, our proposed method, using only uni-modal LiDAR data, outperforms the uni-modal baseline and achieves 97% of the performance of multi-modal 2DPASS. Notable mIoU improvements are observed in small and thin classes, such as fence, motorcycle, and person. Figure 6 and Figure 7 provide the qualitative examples of segmentation results on the SemanticKITTI validation set. The figures show that our method produces more accurate predictions than the baseline and 2DPASS. Specifically, Figure 6 demonstrates that the proposed method achieves more accurate segmentation for thin objects such as tree trunks and fences. Figure 7 illustrates that the proposed method achieves more accurate predictions in challenging cases where confusion may occur within the same super-category. These results demonstrate the effectiveness of our proposed pseudo multi-modal approach in learning 3D representations.

As shown in Table 5, our pseudo multi-modal framework also outperforms the uni-modal baseline and achieves 99% of the performance of multi-modal 2DPASS in the nuScenes dataset. These results provide strong evidence for the scalability and generalizability of our approach. Additionally, Figure 1 visualizes the distribution of average 3D features. The proposed method shows that, compared to the baseline, semantically similar classes are positioned closer together, similar to real multi-modal methods. This observation demonstrates that the proposed method effectively distills semantic information inherent in unimodal data into the LiDAR segmentation network. Table 6 highlights the efficiency of the proposed method, showing that it achieves performance comparable to existing methods while requiring significantly fewer parameters, further supporting the effectiveness and practicality of our approach.

## 5. Conclusions

In this paper, we introduce a pseudo multi-modal approach to LiDAR semantic segmentation. We propose a novel artificial 2D image construction method to create pseudo modal (3D LiDAR, artificial 2D image) pairs from uni-modal LiDAR data. Experiments on the SemanticKITTI and nuScenes benchmarks demonstrate that our proposed pseudo multi-modal approach achieves comparable performance (97–99% mIoU) to real multi-modal methods. The proposed method significantly reduces data acquisition costs during training and computational burden during inference, making it a practical solution for real-world applications.

## Figures and Tables

**Table 1:** Ablation study of text prompt template selection on SemanticKITTI validation set. The class name row in the table indicates the IoU for each respective class. The single default template “A photo of a {label}.” achieves a higher accuracy than the prompt ensembles. We hypothesize that noise from unsuitable templates in the conventional template set adversely impacts the averaged text embedding.

	Default Template	CLIP Templates	MaskCLIP Templates
mIoU	65.7	65.2	65.3
car	96.0	95.6	96.3
bicycle	50.4	50.7	49.6
motorcycle	71.5	76.4	70.3
truck	88.3	77.8	88.6
other-vehicle	64.3	56.8	66.7
person	72.2	74.9	73.6
bicyclist	85.1	89.0	88.8
motorcyclist	0.0	0.1	1.1
road	92.6	93.1	92.4
parking	47.3	45.6	45.8
sidewalk	79.2	79.3	78.4
other-ground	1.2	2.8	4.8
building	91.3	91.1	89.0
fence	66.4	63.1	57.2
vegetation	88.4	88.8	87.2
trunk	70.9	70.5	70.1
terrain	74.7	75.1	71.7
pole	61.9	58.9	59.3
traffic-sign	46.1	50.4	49.5

**Table 2:** Ablation study of class hierarchy information on SemanticKITTI validation set. The class name row in the table indicates the IoU for each respective class. Including hierarchical information in the text prompt template brings semantically similar classes closer in the embedding space, resulting in improved mIoU.

	Base	Base + Sup	Base + Neg
mIoU	65.7	65.7	66.6
car	96.0	96.0	96.3
bicycle	50.4	48.9	50.1
motorcycle	71.5	72.8	74.7
truck	88.3	84.3	89.6
other-vehicle	64.3	65.1	65.5
person	72.2	75.0	76.1
bicyclist	85.1	87.5	90.0
motorcyclist	0.0	0.3	0.3
road	92.6	92.3	93.1
parking	47.3	46.7	48.4
sidewalk	79.2	78.5	79.7
other-ground	1.2	9.7	4.2
building	91.3	89.9	90.9
fence	66.4	58.7	62.1
vegetation	88.4	88.0	88.1
trunk	70.9	71.2	71.3
terrain	74.7	74.8	74.5
pole	61.9	61.0	60.5
traffic-sign	46.1	48.1	49.8

**Table 3:** Ablation study of class descriptions on SemanticKITTI validation set. The class name row in the table indicates the IoU for each respective class.

	Class Name	Synonym Set	Definition
mIoU	65.7	65.8	65.7
car	96.0	96.2	96.4
bicycle	50.4	48.9	48.8
motorcycle	71.5	71.2	74.1
truck	88.3	81.5	77.5
other-vehicle	64.3	63.3	64.2
person	72.2	74.8	76.0
bicyclist	85.1	88.8	89.0
motorcyclist	0.0	2.6	0.0
road	92.6	93.0	93.0
parking	47.3	48.4	47.6
sidewalk	79.2	79.5	79.7
other-ground	1.2	3.1	7.8
building	91.3	91.0	91.0
fence	66.4	61.3	63.5
vegetation	88.4	89.0	88.5
trunk	70.9	71.0	71.3
terrain	74.7	77.0	75.3
pole	61.9	59.5	60.3
traffic-sign	46.1	50.1	50.6

**Table 4:** Quantitative evaluation on the SemanticKITTI validation set. The class name row in the table indicates the IoU for each respective class. † and ‡ indicate the result of the reproduced and pre-trained model, respectively.

Method	Baseline ^†^ [27]	Ours	2DPASS ^‡^ [6]
Modality	LiDAR	LiDAR	LiDAR + RGB
mIoU	63.1	66.6	68.5
car	95.8	96.3	96.8
bicycle	45.7	50.1	52.5
motorcycle	64.0	74.7	76.3
truck	81.5	89.6	90.7
other-vehicle	60.1	65.5	71.3
person	70.5	76.1	78.3
bicyclist	86.3	90.0	92.3
motorcyclist	0.7	0.3	0.0
road	91.8	93.1	93.2
parking	44.8	48.4	50.7
sidewalk	78.1	79.7	80.0
other-ground	0.8	4.2	8.4
building	88.7	90.9	92.2
fence	54.0	62.1	68.2
vegetation	87.6	88.1	88.3
trunk	68.0	71.3	71.1
terrain	74.1	74.5	74.6
pole	58.3	60.5	63.9
traffic-sign	47.6	49.8	53.4

**Table 5:** Quantitative evaluation on the nuScenes validation set. The class name row in the table indicates the IoU for each respective class. † and ‡ indicate the result of reproduced and pre-trained models, respectively.

Method	Baseline ^†^ [27]	Ours	2DPASS ^‡^ [6]
Modality	LiDAR	LiDAR	LiDAR + RGB
mIoU	75.7	77.9	78.0
barrier	75.1	75.8	76.1
bicycle	42.8	48.5	48.4
bus	92.8	95.5	95.1
car	90.7	92.0	91.1
construction-vehicle	47.6	56.3	58.1
motorcycle	83.7	86.3	86.7
pedestrian	77.3	80.1	80.0
traffic-cone	61.0	63.8	63.0
trailer	70.4	71.1	71.2
truck	84.0	86.5	87.0
driveable-surface	95.9	96.3	96.3
other-flat	71.7	71.6	72.9
sidewalk	72.9	74.2	74.2
terrain	73.3	74.1	74.0
manmade	86.9	88.1	88.0
vegetation	84.9	86.2	85.8

**Table 6:** The trade-off comparisons between mIoU and number of parameters (# Parameters) on the SemanticKITTI validation set. The proposed pseudo multi-modal approach achieves comparable performance to previous methods with significantly fewer parameters.

Method	Modality	mIoU	# Parameters
MinkowskiNet [12]	LiDAR	63.1	21.7 M
SPVCNN [27]	LiDAR	63.8	21.8 M
PMF [5]	LiDAR + RGB	63.9	36.3 M
Sphereformer [29]	LiDAR	67.8	32.3 M
Ours	LiDAR	66.6	1.9 M

## Data Availability

The original SemanticKITTI [23] data presented in this paper are openly available at https://www.semantic-kitti.org/, accessed on 22 August 2023.
